# Polyneuropathy and noncompressive cauda equina syndrome as manifestation of systemic lupus erythematosus: a case report

**DOI:** 10.1186/s13256-025-05536-0

**Published:** 2025-09-26

**Authors:** Ahmad Tirmizi Jobli, Sharifah Aishah Wan, Cheng Lay Teh, Yaw Kiet Cheong, Cindy Hui San Kueh

**Affiliations:** 1 Department of Radiology, Faculty of Medicine and Health Sciences, University Malaysia Sarawak, Sarawak, Malaysia; 2 Rheumatology Unit, Sarawak General Hospital, Sarawak, Malaysia

**Keywords:** Systemic lupus erythematosus, Neuropsychiatric lupus, Polyneuropathy, Cauda equina syndrome, Case report

## Abstract

**Background:**

Neuropsychiatric systemic lupus erythematosus covers a wide range of central nervous system and peripheral nervous system conditions. We report a rare case of a man with progressive muscle weakness and cauda equina syndrome.

**Case presentation:**

A 32-year-old man of Chinese ethnicity presented with a 2-month history of progressive muscle weakness of the upper and lower limbs, associated with inflammatory joint pains, and rashes over the upper and lower limbs. Examination showed post-inflammatory hyperpigmentation over the upper and lower limbs and weakness in the proximal muscles of the upper and lower limbs. Laboratory investigations showed anemia, leukopenia, lymphopenia, positive antinuclear antibodies, positive anti-double-stranded deoxyribonucleic acid antibodies, positive anti-Smith antibodies, and positive anti-ribosomal P protein antibodies. He later developed acute urinary incontinence and weak anal tone. Magnetic resonance imaging of the spine showed leptomeningeal enhancement of the cauda equina. He was diagnosed with neuropsychiatric lupus with peripheral polyneuropathy and cauda equina syndrome. His condition was complicated with an infection and pulmonary embolism, but he eventually recovered with treatment. He was treated with high-dose steroids, hydroxychloroquine, intravenous immunoglobulin, anticoagulation, cyclophosphamide, and subsequently, mycophenolate mofetil.

**Conclusion:**

This case highlights the rarity of peripheral neuropathy and noncompressive cauda equina syndrome in systemic lupus erythematosus. A collaborative effort by clinicians in multiple fields is required to achieve a diagnosis and satisfactory treatment in this patient.

## Background

Neuropsychiatric systemic lupus erythematosus (NPSLE) covers a broad range of clinical presentations and is often challenging to diagnose and manage. It encompasses central nervous system and peripheral nervous system manifestations, which were described in the 1999 American College of Rheumatology definitions for NPSLE [1]. The estimated NPSLE prevalence ranges from 14% to 95%, and this wide range is due to the variability in clinical presentation, different selection criteria, and heterogeneity in the population studied [2]. Accurate diagnosis of NPSLE integrates history-taking, thorough physical examination, laboratory investigations, neuroimaging, and electrophysiology studies.

The diagnostic challenge is especially obvious when the patient does not have common systemic lupus erythematosus (SLE) features. We report a rare case of NPSLE in a man with progressive limb weakness and urinary incontinence.

## Case presentation

This case describes a 32-year-old male of Chinese ethnicity with a history of progressive bilateral upper and lower limb muscle weakness 2 months prior to presentation. It initially involved the proximal muscles of the lower limbs, and the patient had difficulty squatting, which progressed to difficulty in standing up and subsequently in lifting his arms. There was associated inflammatory joint pain over the fingers and wrists, rash over the bilateral lower limbs, loss of appetite, and loss of weight. There was no history of trauma, back pain, prolonged fever, facial rash, photosensitivity, oral ulcers, alopecia, chronic cough, hemoptysis, or urinary or bowel incontinence. There was no past medical history and no significant family history. He was a cigarette smoker and worked as a delivery driver.

Physical examination showed multiple postinflammatory hyperpigmentation over the bilateral upper and lower limbs. The rashes were flat, nontender, and dark brown in color and measured 1 cm × 2 cm with distinct borders. The rashes were situated at various regions on the forearms and lower legs. There was no facial rash, photosensitivity, or oral ulcers. Cardiovascular, respiratory, and abdominal examinations were normal. Neurological examination revealed no muscle fasciculations, and he had normal tone and reflexes bilaterally. There was proximal myopathy with bilateral shoulder power 3/5, bilateral elbow power 3/5, bilateral wrist power 5/5, and bilateral hand grip power 5/5. In the lower limbs, the bilateral hip power was 3/5, bilateral knee power was 5/5, and bilateral plantar flexion and dorsiflexion was 5/5. The sensation was normal in all dermatomes. Bilateral Babinski signs were downgoing, proprioception was normal, and the cerebellar signs were negative.

The initial blood investigations showed a full blood count with anemia, leukopenia, and lymphopenia. The hemoglobin was 11.6 g/dL, total white blood cell count 2.96 × 10^3^ /µL, lymphocyte count 0.92 × 10^3^ /µL, and platelet count 148 × 10^3^/µL. Renal profile was normal with sodium at 130 mmol/L, potassium at 3.5 mmol/L, urea at 2.70 mmol/L, and creatinine at 45 µmol/L (normal 62–106). Creatine phosphokinase was normal at 47 U/L (normal 39–308). Liver function test was normal. Serum cortisol was normal at 512 nmol/L (normal 133–537). Thyroid function test was normal, with free T4 at 18.80 pmol/L (normal 13.10–21.30) and thyroid-stimulating hormone (TSH) at 2.550 mIU/L (normal 0.300–3.180). C3 was low at 0.32 g/L (normal 0.90–1.80), and C4 was low at 0.07 g/L (normal 0.10–0.40). Antinuclear antibody (ANA) test was positive at > 200 CU, and anti-double-stranded deoxyribonucleic acid (anti-dsDNA) antibodies were positive at 50.6 IU/mL (< 27 negative). Anti-Smith (anti-Sm) antibodies were positive at 125.7 CU (< 20 negative), anti-ribonucleoprotein (anti-RNP) antibodies were positive at 21.5 CU (< 20 negative), anti-Ro-60 antibodies were positive at 21.7 CU (< 20 negative), and anti-ribosomal P (anti-Rib-P) antibodies were positive at > 362 CU (< 20 negative). ANA, anti-dsDNA, and extractable nuclear antibody (ENA) tests were performed with chemiluminescent immunoassay. Nerve conduction studies and electromyography tests showed axonal sensorimotor polyneuropathy and irritative myopathy. The patient declined a cerebrospinal fluid examination. He was diagnosed with systemic lupus erythematosus on the basis of the following clinical features: constitutional symptoms (loss of appetite and loss of weight), postinflammatory hyperpigmentation over the upper and lower limbs, peripheral polyneuropathy, leukopenia, lymphopenia, anemia, low C3 and C4, and positive ANA, anti-dsDNA, anti-Sm, anti-RNP, and anti-Rib-P antibodies. He was started on high-dose steroids and hydroxychloroquine.

**Fig. 1:**
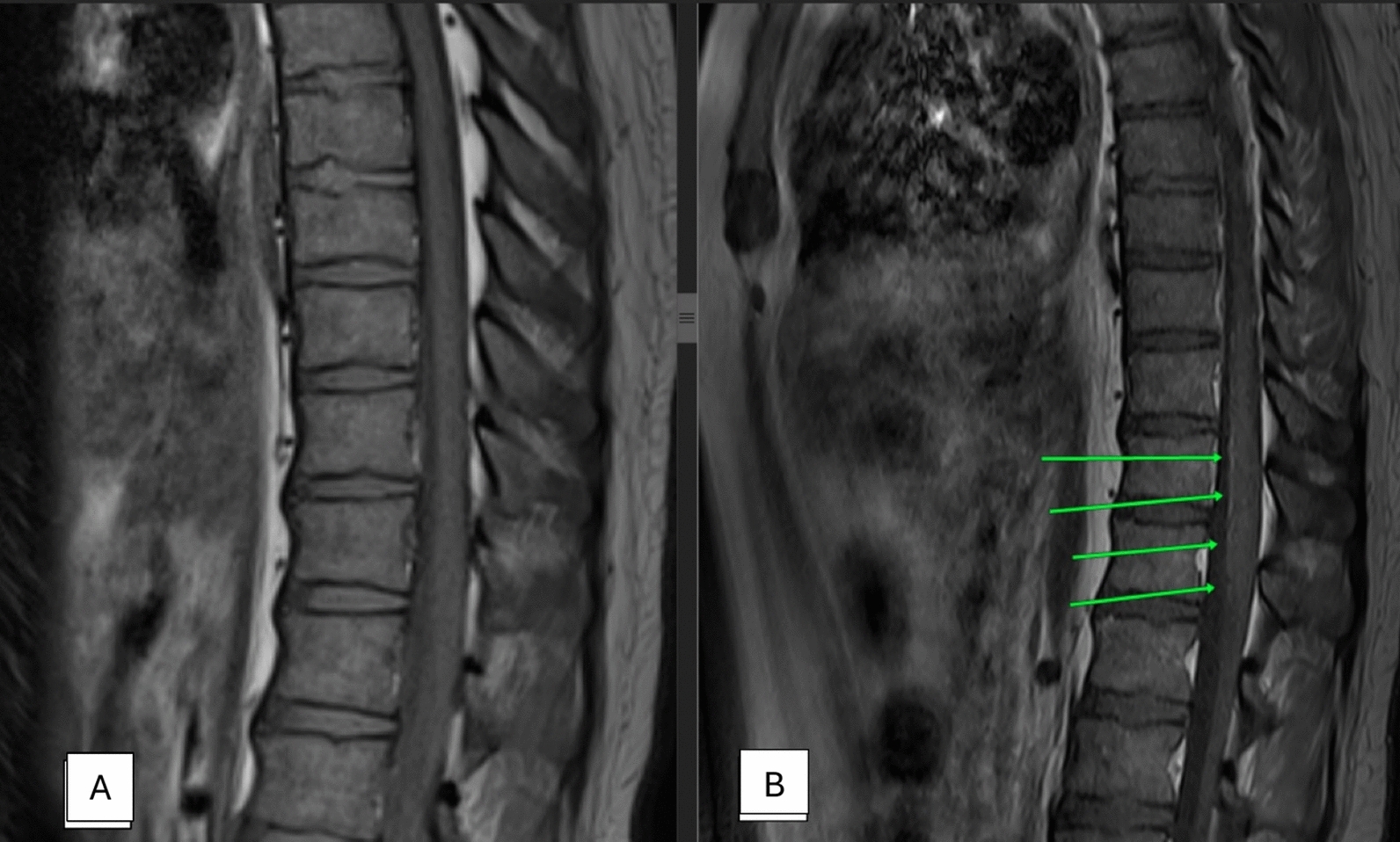
Image **A**: Pre-contrast. Image **B** demonstrates abnormal leptomeningeal enhancement (green arrow) seen post contrast. The underlying cord is normal with no abnormal signal

**Fig. 2:**
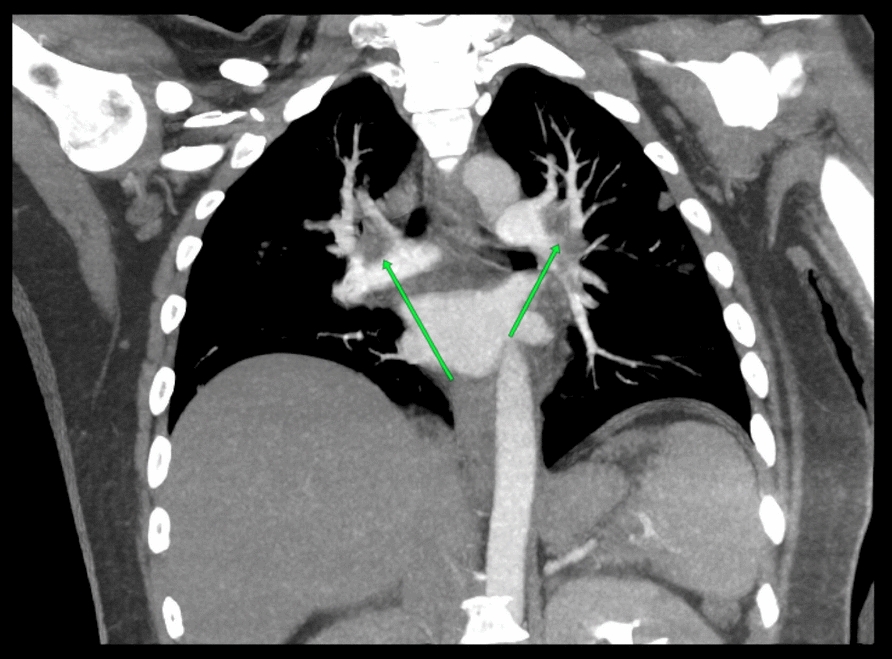
Filling defect in both segmental branches of the pulmonary arteries (green arrows)

After 2 days of admission, he complained of urinary incontinence. The patient’s anal tone was weak, and his perianal sensation was reduced. Cauda equina syndrome was suspected, and a magnetic resonance imaging (MRI) scan of the spine showed leptomeningeal enhancement at the caudal part of the spinal cord (Fig. 1), with no cord compression, indicating that cauda equina syndrome was due to an inflammatory process of the caudal part of the spinal region. He was treated with pulse intravenous methylprednisolone, intravenous immunoglobulin (IVIG) and intravenous cyclophosphamide infusion. He also underwent physiotherapy and rehabilitation. His total white blood cell and platelet count improved 3 days after starting pulse methylprednisolone. His muscle weakness remained the same despite the pulse methylprednisolone, intravenous immunoglobulin, and intravenous cyclophosphamide at the time. After 2 weeks of admission, he developed fever and coughing with a small amount of sputum. A chest x-ray showed multiple consolidations in the bilateral lungs. A computed tomography (CT) of the thorax showed bilateral cavitating lung lesions. Blood culture grew *Staphylococcus aureus* sensitive to cloxacillin, and he was started on a 6-week course of intravenous cloxacillin. A transthoracic echocardiography was performed and did not show any valvular endocarditis. At week 3 of admission, he complained of chest pain with hypotension and tachycardia. Electrocardiography (ECG) showed tachycardia with T inversions over V2-6. D-dimer was positive at > 0.2 µg/mL (normal < 0.2). A computed tomography pulmonary angiogram (CTPA) showed pulmonary embolism of bilateral distal main pulmonary arteries extending into the lower lobe segmental branches (Fig. 2). He underwent an ultrasound-assisted, catheter-directed thrombolysis (EKOS) for the pulmonary embolism. He was started on a low-molecular-weight heparin and later warfarin. Lupus anticoagulant and anticardiolipin antibodies were negative. Anti-β2-glycoprotein IgG antibodies were positive at 33.9 CU (< 20 negative). The combination of pulmonary embolism and a positive anti-β2-glycoprotein antibody test led to the diagnosis of secondary antiphospholipid syndrome.

Mycophenolate mofetil was started in week 5 of admission. At week 6 of admission, there was only mild improvement in his muscle power. He continued his rehabilitation in another facility for another 4 weeks and eventually regained full muscle power and full mobility with no further urinary symptoms at 10 weeks after the initial admission. His current treatment includes steroids, hydroxychloroquine, mycophenolate mofetil, and warfarin. He remained well during follow-up.

## Discussion

This case highlights the difficulties in diagnosing and managing SLE in a patient with uncommon clinical manifestations. His clinical features at presentation were abnormal rash, peripheral polyneuropathy, cauda equina syndrome, leukopenia, and anemia. ANA, anti-dsDNA, anti-Sm, and anti-β2 glycoprotein were later found to be positive. The diagnosis was delayed as he lacked the typical malar rash, oral ulcers, alopecia, and glomerulonephritis commonly seen in other patients with SLE.

His initial neurological presentation was that of muscle weakness of bilateral shoulders, elbows, and hips, with normal tone, reflexes, and sensation. The differential diagnoses for this neurological presentation were acute inflammatory demyelinating polyradiculoneuropathy, autoimmune inflammatory myositis, endocrine disorders such as hypothyroidism and Cushing’s syndrome, hereditary myopathies, and neuromuscular junction disorders such as myasthenia gravis. However, the other clinical features associated with the other differential diagnoses were absent. As is often the case, a comprehensive review of the clinical features and additional investigations will lead to the correct diagnosis. In this case, the nerve conduction studies confirmed the presence of peripheral polyneuropathy.

NPSLE has a variety of manifestations, which include central nervous system (such as aseptic meningitis, cerebrovascular disease, myelopathy, seizures, acute confusional state, cognitive disorders, and psychosis) and peripheral nervous system (such as acute inflammatory demyelinating polyradiculoneuropathy, mononeuropathy, cranial neuropathy, myasthenia gravis, and polyneuropathy) manifestations [1]. This case is unusual in that he had both peripheral polyneuropathy and noncompressive cauda equina syndrome, both related to inflammation present in NPSLE. Cauda equina syndrome was not widely described in NPSLE literature. In our literature review, there were only five case reports that featured polyneuropathy and cauda equina syndrome in patients with SLE [3–7].

Radiological imaging is an important diagnostic tool in NPSLE. It helps to define the clinical features and rule out other diagnoses. In our case, during the initial presentation, it was important to distinguish between polyneuropathy, vasculitis, idiopathic inflammatory demyelinating disorders, inflammatory transverse myelitis, and paraneoplastic syndrome. An MRI of the spine in this case showed leptomeningeal enhancement at the cauda equina region, with no features of cord compression, transverse myelitis, or multiple sclerosis. There were also no features of malignancy in the MRI and subsequent CT scan. Other causes of cauda equina syndrome are infection, inflammation, and neoplasm [9].

This patient’s admission was complicated by *Staphylococcus aureus* infection and pulmonary embolism. He was diagnosed with antiphospholipid syndrome secondary to SLE due to the presence of pulmonary embolism and a positive anti-β2-glycoprotein antibody. The 2023 American College of Rheumatology/European League against Rheumatism (ACR/EULAR) antiphospholipid syndrome classification criteria [8] require an entry criteria of one clinical criterion (macrovascular thrombosis (venous or arterial), microvascular thrombosis, obstetric, cardiac valve, and hematology) and one positive antiphospholipid antibody (lupus anticoagulant, anticardiolipin antibody by solid phase assay (enzyme-linked immunosorbent assay (ELISA)) or anti-β2-glycoprotein antibody by solid phase assay (ELISA)). However, the only antiphospholipid antibody laboratory test available in our center was the chemiluminescent immunoassay and not the enzyme-linked immunosorbent assay (ELISA). Even though he did not strictly fulfill the classification criteria for antiphospholipid syndrome, we diagnosed and treated him for pulmonary embolism due to secondary antiphospholipid syndrome with SLE on the basis of his overall clinical presentation and the additional investigations.

The final diagnosis of SLE with secondary antiphospholipid syndrome was made after taking into account the various clinical manifestations (inflammatory joint pains, abnormal rash, peripheral polyneuropathy, cauda equina syndrome, pulmonary embolism), blood investigations (leukopenia, lymphopenia, anemia, low complements), serological investigations (positive ANA, anti-dsDNA, anti-Sm, anti-ribosomal P protein, anti-β2-glycoprotein antibody), nerve conduction studies, and radiological imaging (leptomeningeal enhancement of cauda equina region). The rarity of peripheral polyneuropathy and cauda equina syndrome in SLE did make the diagnosis challenging, but the significant improvement after treatment with steroids and other immunosuppressants was reassuring, despite other complications such as infection and pulmonary embolism that followed.

## Conclusion

This case highlights that SLE may present with atypical and rare clinical manifestations. A collaborative effort in a multidisciplinary team of rheumatologists, neurologists, and neuro-radiologists is often helpful to establish the diagnosis and treatment plan in complex cases such as the one we encountered in this patient.

## Data Availability

The clinical data and images are available from the corresponding author upon request.

## Abbreviations

NPSLE: Neuropsychiatric systemic lupus erythematosus
SLE: Systemic lupus erythematosus
ANA: Antinuclear antibody
Anti-dsDNA: Anti-double-stranded deoxyribonucleated acid antibody
Anti-Sm: Anti-Smith antibody
Anti-RNP: Anti-ribonucleoprotein antibody
Anti-Rib P: Anti-ribosomal P protein antibody
